# A mHealth-based nursing model for assessing the health outcomes of the discharged patients with nasopharyngeal carcinoma: a pilot RCT

**DOI:** 10.1186/s12912-022-00993-0

**Published:** 2022-08-01

**Authors:** Tingting Liao, Liyan Qiu, Jingwen Zhu, Jiayan Li, Yanxin Zhang, Li Yang

**Affiliations:** Department of Nursing, The First Affiliated Hospital of Guangxi Medical University, Nanning, Guangxi China

**Keywords:** mHealth, Nasopharyngeal carcinoma, Nursing model, Health outcomes

## Abstract

**Background:**

Nasopharyngeal carcinoma (NPC) is one of the most common head and neck malignancies, having a high incidence in Guangxi, China. Although chemoradiotherapy offers more effective cancer treatment, it also causes a variety of acute and chronic side effects, seriously affecting the quality of life. NPC has evolved into a chronic disease with most patients opting for home-based rehabilitation. Therefore, efforts on improving the home-based extended care services to improve the quality of life of patients are booming. The Chinese government encourages the use of internet technology for expanding the prospect of nursing. This study aimed to evaluate the impact of a mHealth-based care model on the health outcomes of discharged patients with nasopharyngeal carcinoma.

**Methods:**

An experimental design was applied for this study. The study enrolled 116 discharged patients who were re-examined in the Radiotherapy Department of the First Affiliated Hospital of Guangxi Medical University from November 2019 to February 2020. These patients were randomized into control and intervention groups (*n =* 58 per group), but during the implementation of the project, there was one dropout in the control group due to the loss of follow-up, and one dropout in the intervention group due to distant metastasis. In the end, 57 patients in the control and intervention groups completed the trial. The control group was subjected to routine discharge guidance and follow-up, while the experimental group was implemented with a mobile health (mHealth)-based continuous nursing intervention model. The scores of the side effects, cancer fatigue, and quality of life were compared between the two groups of patients for 3, 6, and 12 months, respectively after discharge from the hospital.

**Results:**

This study included 114 patients and there were no significant differences in the baseline data between the two groups. After 6 and 12 months of intervention, the severity of radiation toxicity and side effects, the scores of cancer-related fatigue, and quality of life (symptom field) of the patients in the interventional group were significantly lowered statistically compared to those in the control group.

**Conclusion:**

This study is based on the mHealth continuous nursing intervention model, which can reduce the side effects of radiotherapy and cancer fatigue, and improve the quality of life.

**Trial registration:**

This study was retrospectively registered as a randomized controlled trial in the Chinese Clinical Trial Center. Registration Date: January 12, 2021, Registration Number: ChiCTR2100042027.

## Introduction

Nasopharyngeal carcinoma (NPC) is a head and neck malignant tumor originating from the nasopharyngeal epithelial cells [1]. The highest ASIRs per 100,000 were in East and Southeast Asia [2]. NPC shows high incidence in Guangxi of China, the annual crude incidence is 30–80/ 100,000 [3, 4]. The incidence of NPC is higher in males than that in females, with a ratio being 2.5 in China in 2015 [5]. The definitive diagnosis is done by the endoscopic-guided biopsy of the primary nasopharyngeal tumor. Nasopharyngeal carcinoma is highly sensitive to ionizing radiation, radiotherapy combined with chemotherapy is the mainstay treatment modality. Although advanced NPC might choose targeted therapy or immunotherapy, local surgery serves as an option for treating locally recurrent NPC [6]. Chemoradiation is still recognized as the best treatment. The present 5-year overall survival rate in an NPC patient with such combinatorial treatment exceeds 80% [7].

However, chemoradiotherapy not only kills the tumor cells but also inevitably damages the normal tissues in the vicinity of the radiation field area, resulting in acute and chronic radiation injury symptoms such as the oropharyngeal acute mucosal injury, dry mouth, taste change, difficulty in opening the mouth and swallowing [8, 9]. These long-term toxicities and side effects syndrome tend to exist for a long time in the patients during the home rehabilitation period even after the termination of the treatment. It is during this post-treatment period that the body’s symptoms accumulate and the negative emotions cooperate and reinforce each other, thereby aggravating the burden of cancer on the patients [10]. In addition, the non-standard follow-up management, as well as the lack of access to healthcare information, seriously jeopardize the quality of life (QOL) of the NPC patients outside the hospital. Therefore, there is an urgent need for systematic and effective continuous nursing services, which would provide a series of long-term professional guidance for promoting changes in attitude and behavior, including paying more active attention to self-health problems and improving compliance with medication. These changes might enable the patients to carry out effective self-care at home [11, 12].

Continuous care is considered one of the essential elements of high-quality health services [13]. It refers to the systematic, continuous, coordinated, and professional health care behavior that is conducive to the home-based rehabilitation of the patients in the unusual medical service institutions or the same medical service institutions under different conditions [14]. China has established a continuous nursing mode, making initial progress in the field of chronic diseases based on the lessons drawn from the overseas extended service modes and combining them with the characteristics of the domestic medical resource system. For example, the 4C extension service mode and effect evaluation scheme has been constructed in Hong Kong and Taiwan [15, 16]. In mainland China, continuous nursing services are widely provided via telephone [17] or platforms such as Tencent QQ, WeChat [18], mobile health apps [19], and other media. These platforms can help in continuing the nursing of patients in the future, and also expose the limitations of the current extended nursing service teams and intervention programs in China, like (1) The diversified and single forms of continuous nursing exist side by side, while each form of continuous nursing is fraught with limitations [20, 21]; (2) Presently, the hospital discharge follow-up and community-based primary health care services constitute the common modes of implementing continuous care in China [20]. (3) About 93.75% of the patients wish to receive extended care services and on-site services after discharge [22], however, the continuous care link in hospitals is relatively weak, and the contradictions between the supply and demand of the extended nursing services are very prominent due to limitations such as uneven distribution of medical resources and insufficient development of communities.

Therefore, this study mainly focused on optimizing the therapeutic resources to help hospitals in providing the patients with high-quality, effective, convenient, and rapid continuation of nursing services for ameliorating the current situation of unbalanced development of medical resources.

Mobile health(m-Health) uses emerging mobile apps and network technologies for healthcare. The robust development of the mobile internet has solved the issue of hospital-based continuous care [23]. The robust development of the mobile Internet has solved the issue of hospital-based continuous care [23]. The 49th report of the China Internet Network Information Center in 2022, as of December 2021 stated the network penetration rate to reach 73.0%, and the proportion of using mobile phones connecting to the Internet to be as high as 98.6%, maintaining a benign growth trend. The WeChat public platform is presently the most widely used internet platform. It is extensively used for the hospital health information release, online appointments, disease intervention, and other fields and can realize the comprehensive interaction of text, pictures, and sounds [24, 25]. Mobile medical services similar to WeChat public platforms are also widely used abroad. For example, the Cancer net Mobile app has been recognized by many oncology experts in the world and has functions of voice or video, text, picture, content or information sharing, internet access, and so on. It provides out-of-hospital care such as treatment, medication, complication management, and so on [26]. However, the WeChat platform (mHealth) of this study was constructed with the help of WeChat software, which is the most popular communication platform in China and has a wide range of audiences, similar to WhatsApp and Kakao Talk. Therefore, the use of WeChat is simpler and more convenient.

The 2016–2020 China nursing development planning document also encourages the use of mobile network technology for improving the level of hospital informatization, extending nursing services to the communities and families, and providing one-stop flexible extension services for the discharged patients to meet their health needs [27]. To date, mobile medical applications have been widely used in the care of cancer patients abroad. Researchers in Denmark have developed a mobile health (MHealth) app for providing continuous care to adolescent cancer patients [28]. Similarly, American researchers have designed a Cancer Survivor Profile-Breast Cancer (CSPro-BC) app to meet the information support needs of breast cancer survivors [29]. These studies at home and abroad have been shown that the efficient and convenient use of the internet and mHealth-based management systems can overcome limitations of time and space in traditionally implementing continuous nursing, accelerating the dissemination of health information, improving the enthusiasm of the individual behavior and user experience, and thus, enable the patients to achieve a higher quality and effective management [30, 31].

Therefore, the goal of this study was first to use the internet technology based on the WeChat public platform to build a mHealth platform for patients with NPC. Secondly, we aimed to investigate the impact of the mHealth-based continuation of care on health outcomes in terms of radiation therapy, fatigue, and QOL of discharged patients with NPC.

## Methods

### Study design

This study is an exploratory pilot study based on a randomized controlled trial lasting from November 2019 to February 2021.

### Patients

The study involved NPC patients who were recruited after reviewing in the Radiotherapy Department of the First Affiliated Hospital of Guangxi Medical University from November 2019 to February 2020.

#### Inclusion criteria

(1) The patients diagnosed by pathology and who have completed chemoradiotherapy; (2) Those aged 18–65 years; (3) With no previous history of mental illness and serious disturbance of consciousness; (4) With an educational level of primary school or above; (5) Having the ability and condition of using the Internet; (6) Were informed and voluntary participants.

#### Exclusion criteria

(1) The patients with distant metastasis or recurrence; (2) Those with language communication and hearing impairment; (3) Combined with other serious diseases or organic malignant tumors.

#### Dropout criteria

(1) Included the dropout and non-compliance; (2) Recurrence, metastasis, or death.

### Sample size calculation

According to the sample size calculation formula involving the comparison of the two sets of means, the sample size was calculated after consulting the literature, such that the two-sided α = 0.05, β = 0.10, and the proportion of the two groups of samples is the same, that is, r = 1. After preliminary experiments, the σ = 4.98，n_c_ = 50 cases were calculated taking into account the 10–15% loss to follow-up rate, with n_c_ = 58 cases, according to the average and standard deviation of the total score of cancer fatigue after 3 months of intervention for the two groups of patients. There were 58 cases in the control group. Therefore, a total of 116 cases were required.

The formula used is as follows:

$${n}_c=\frac{\left(r+1\right)}{r}\frac{\sigma^2{\left({Z}_{1-\alpha /2}+{Z}_{1-\beta}\right)}^2}{{\left({u}_t-{u}_c\right)}^2}$$

where r is the sample ratio of the two groups, u_t_ and u_c_ are the mean values of the two groups, σ2 is the combined variance, and n_c_ is the sample size of the control group.

### Randomization, assignment, and blinding

In this study, the IBM SPSS STATISTICS 22.0 random number-generation method divided the patients into the experimental and control groups, with 58 cases each (The IBM SPSS STATISTICS 22.0 software is a professional data statistical analysis software developed and continuously updated and improved by Norman H. Nie, C. Hadlai (Tex) Hull, and Dale H. Bent). Randomization was generated by an investigator who was not involved in data collection or intervention. After the participants expressed their interest in the research, the research recruiters used the IBM SPSS STATISTICS 22.0 software for randomized allocation. The researchers and the patients were blinded to the group they would be assigned to. The allocation ratio of the intervention to control group subjects was 1:1.

The nature of the intervention implied that blinding of the patients was not possible, albeit the patients were blinded to the study hypotheses. The blinding of the assessors and analysts were achieved, as the participants’ allocation was concealed from the assessors and the statistical analysis team. All data were registered in an anonymous database by the patients’ identification number.

### The clinical teams

(1) Composition of the nursing team: The chief leader was the director of the radiotherapy department and the director of the nursing department, who was responsible for the operation of the whole team; the deputy leader was the head nurse, who assisted the whole team to carry out nursing. The members included 5 nursing staff of the radiotherapy department, 2 attending physicians, 1 graduate student, 1 dietitian, and 1 network engineer, with clear responsibilities assigned. (2) The team training: the training content involved the system platform and operation platform, the use and maintenance, data collection and input, the continuation of the nursing intervention methods, specialized nursing knowledge and techniques of the centralized and unified training, to ensure that the research and control the consistency of the implementation of prejudice, ensuring that every member is the master for platform operation, problem handling, and answering questions in the process of diagnosis and prognosis. (3) The responsibility and role allocation: the nursing members were responsible for the establishment of the patients’ health records, formulation of the discharge plan, knowledge push, answer questions. The doctors participated in the formulation of follow-up plans for patients discharged from the hospital and answered questions online.

### Intervention measures

Control group: There was routine discharge guidance and follow-up by the department follow-up staff every 3 months through telephone follow-up and out-patient follow-up guidance for understanding the side effects of the radiotherapy in the patients after discharge, and providing diet, oral care, functional exercise, follow-up, and other instructions. Experimental group: Based on the mobile health care model. Detailed as follows:

### Search strategy

To learn from for building a better mobile medical platform and intervention plan based on good experience and results, an extensive literature search was performed considering both published English-language or Chinese-language reports for systematic searches in each of six main databases: VIP, CNKI, CBM, PubMed, Web of Science, and Embase. The search strategy for this study included a combination of keywords that were meant for capturing all the possible relevant studies related to mHealth and cancer. Both the descriptor-based and more general searches were conducted. The following search string was used: (Mobile applications ‘OR’ Smartphone applications ‘OR’ mHealth) ‘AND’ (Cancer ‘OR’ Neoplasms) followed by further searching using specific outcome measures: (‘morbidity’ OR ‘mortality’ OR ‘quality of life’ OR ‘hospital. The relevant literature retrieved was summarized and analyzed for providing a reference for this research.

### Construction of the mobile health (mHealth)-platform

m-Health was developed by a unified team of experts comprising those from the Radiation Oncology Department of the First Affiliated Hospital of Guangxi Medical University and an information technology company. Based on the needs of the patients after discharge from the hospital, the characteristics of recovery, and the use of “Internet +” media technology, the research, and development was simple, and fully functional mobile health (mHealth) platform without installation. The platform architecture involved a system management platform and an operating platform. The system management platform involved functions, such as information management, data management, and platform maintenance. The operating platform: ran in the form of the “WeChat Mini Program.” ①Patient side: with functions of health information base, online consultation, data upload, personalized management, and health file; ②Medical care side: with functions of high-quality nursing resource sharing module, Patient library, Online clinic and Doctor information. Moreover, as for evaluating the usability test of the application, we conducted a questionnaire survey on the patients in the early stage, including the quality of platform resources and platform system，the evaluation of system quality, platform service quality and user experience indicates that patients can achieve above medium level evaluation. The details are as follows:

#### Service platform

##### A patient side

**Fig. 1:**
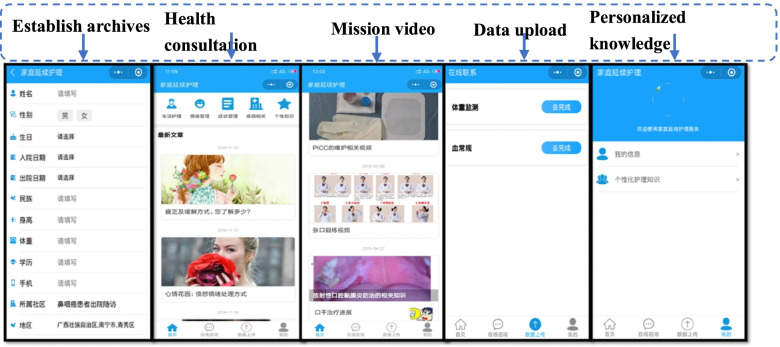
Patient side

1) Common interface: ①Knowledge database: it includes 5 types of knowledge for the patients to read and browse; ②Online consultation module: the patients can interact with the medical staff online to consult the basic knowledge of the disease, personalized dietary guidance, symptom management, emotional management, medication guidance, and other issues; ③ Data upload module: used for collecting the patient’s weight, blood routine, and other relevant health data, to facilitate the medical staff to timely grasp the patient’s recovery. 2) Personal interface: My health files module: includes the basic information about the patients. Personalized demand knowledge module: the nursing team customized the personalized discharge plans and health education plans for the patients according to their nursing problems, complications assessment, and needs, and regularly sent to the patients for 2 weeks **(**Fig. 1**).**

##### B medical care side

**Fig. 2:**
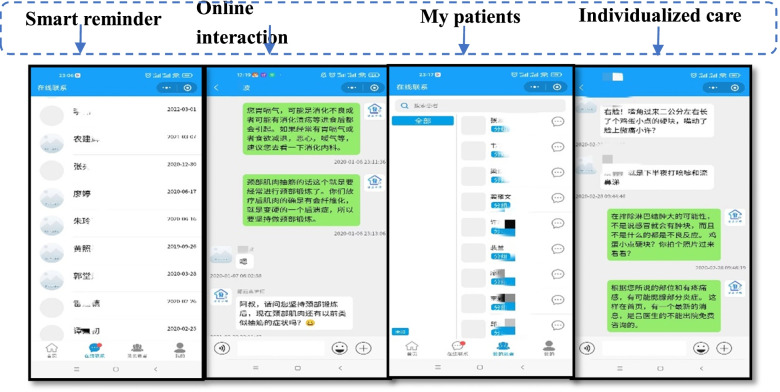
Medical service terminal

The members of the continuous care team carried out the continuous care services through the functional modules customized on the medical care side. The medical side includes ① My patient database managed the patients who entered the home care platform, the patient’s name, height, contact information, personal contact information, whether allergic drugs, and other information could be checked. ② Online Q&A carried out the basic continuous care services, such as online q&A, providing personalized guidance and health-related knowledge, operation guidance, reminder reading, and other follow-up content. ③ The high-quality nursing resource sharing module: available on the public interface, the activities that could be browsed, technology development, and high-quality clinical nursing resources of our hospital, enriched the professional technical knowledge and improved the professional level (Fig. 2).

**Fig. 3:**
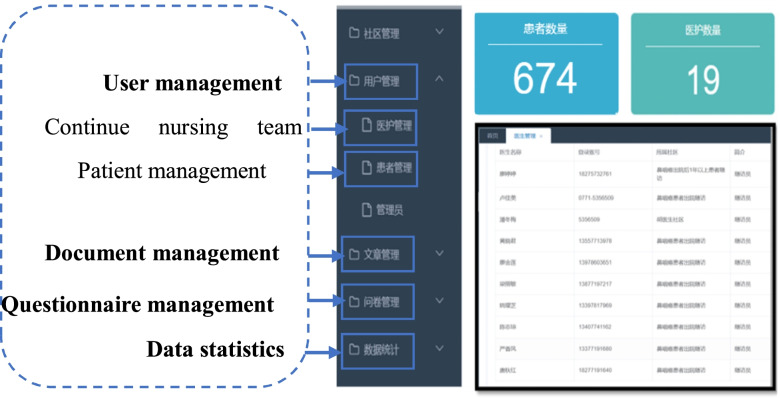
System management platform

**(2)** System management platform: It has five management modules: community management (the discharged patients in different regions), user management (medical care management and patient management), article and video, questionnaire, and statistical data management. The platform maintenance, and management, editing, updating, and sending of healthcare information, questionnaire pushing, data statistical analysis, and other functions can be carried out according to the five management modules (Fig. 3).

### System security policy

The security of the platform: (1) Signing the confidentiality contracts with the technology developing companies to avoid breach of the patient privacy; (2) The use of cloud computing server, virtual firewall, role permission control, and other security operations. In addition, the managers should sign the relevant confidentiality agreements for software development, data transmission, and patient management for protecting patient privacy.

### Application security

The platform has both the function of authority and security management: hence, two levels of permissions are set in the medical terminal. The first level of authority is the super administrator responsible for managing the account and password of the team members, the setting of patients’ admission, as well as the operation and maintenance of the platform. The team members can join the medical terminal only after the super administrator grants the invitation code, and it is involved in the micro signal. To ensure security, others are barred from logging in to the medical terminal using their account. The second level of authority is involved those of the nursing team members responsible for regularly releasing video and audio health education materials, and questionnaires, and answering the questions of the patients. The patients and the families are only limited to using their rights. With the help of the security function of the WeChat software account management, information security was ensured since other accounts can’t be logged in without authorized permission.

### Data access security

The platform was bound to a WeChat account and needed registration so that other accounts were barred from logging in ensuring the confidentiality of patient information; patients and families were only limited to use the rights and could only access the functions and contents that should be seen within their authority.

### The intervention group

#### Before discharge

**Fig. 4:**
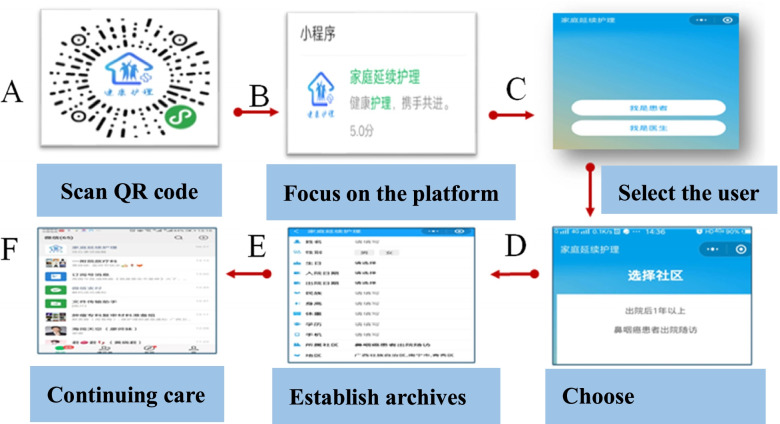
Access platform flow chart

The patients in the intervention group registered and entered the platform through the QR code scanned by Wechat, and the nurses helped them to establish their electronic health records, including their names, home addresses, mobile phone numbers, disease diagnoses, discharge times, current weights, blood routines followed by the existing symptoms and current nursing needs. The operation of the platform was simple and easy to understand, which ensured that the patients would be proficient in using all the functions (Fig. 4).

#### After discharge

The intervention group implemented mHealth based continuous nursing intervention on the basis of routine nursing methods of the control group, as follows:

##### Individualized assessment of the health problems of the patients

This step included nutrition, sleep, exercise, and psychology. The specialist assessment included nasal congestion, hearing loss, dry mouth, difficulty in opening the mouth, radioactive decay, and other toxic side effects. This step provided the basis for compiling the knowledge base of health information and personalized nursing in the later period.

##### Establishment of the knowledge base of health information

It was established based on literature data and the existing understanding of the problems and needs of the patients. The content of the knowledge base was edited by the Continuing Care team using pictures and videos to ensure comprehension in a readable and professional manner. The health information knowledge base included the following four health information modules: “Life Care”, “Symptom Management”, “Emotion Management”, and “Basic Knowledge of Diseases”. Moreover, the content of each knowledge base was dynamically adjusted to the number of questions raised by the patients, changing trends of adverse radiation reactions, negative emotions, nursing needs, and other factors. The content of each information knowledge base was scientific and comprehensive, which met the needs of patients at all levels after their discharge.

##### Upload health information on the platform

According to the established health information knowledge base, the nursing team uploaded on the platform every 2 weeks in a planned and purposeful manner. The uploaded content was in the form of pictures, text, and videos. The patients could browse on the patient side according to their needs. The platform included a message reminder function, when the nurses uploaded the latest health information, they reminded the patients to read the relevant health information.

##### Personalized care

**1)** According to the results of the individualized assessment, the symptoms, psychology, nutrition, and sleep problems of the patients were analyzed. The initiative to answer and guide online with words, pictures, and voices was taken, and the corresponding knowledge from the health information knowledge base was selected and sent to the patients on time, which facilitated the patients’ perspective of their health and encouraged them to imbibe effective healthcare behaviors.

**2)** Online interaction: the patients could browse the rehabilitation knowledge on “life care, disease knowledge, emotion management, symptom management” in the “health information module” of the “patient side”; when in doubt, they could consult the medical staff about the nursing knowledge by using the “Online consultation” module. The team members could click to view the message to timely answer the patient’s question and provide guidance or advice or handle emergency needs.

**3)** By clicking on the “My Patient” module, the continuous nursing team urged and instructed the patients to clock in and upload the exercise videos for functional exercises in cases with difficulty in opening the mouth and neck fibrosis, every week to ensure the correctness of the nursing behaviors.

**4)** The patients were reminded to browse the latest published knowledge, monitor the reading volume of the patients in the background, and encourage and urge patients to actively and timely browse through the learning materials and self-learn.

**5) Patient-related data upload:** This step reminded the patients to upload their weight and blood indices results by clicking on the “My” module, which was convenient for the medical staff for consultation and guidance purposes.

**(5) Phased evaluation:** The toxicity and side-effects questionnaire, QOL scale, and cancer-related fatigue (CRF) scale were employed to collect and evaluate the patients at 3, 6, and 12 months after the intervention, and the intervention scheme was continuously and dynamically optimized, as required.

### Instruments

#### General situation questionnaire

It included the general baseline data (gender, age, occupation, education level, etc.) and disease data.

#### Toxic side effects of radiotherapy

It was evaluated by the oncology Radiotherapy and RTOG Acute Radiation Injury Classification Standard [32]. The specific rating criteria are as follows:

(1) Dry mouth: grade 5, Grade 0: no change; Grade 1: slight, slightly dry mouth during night sleep; Grade 2: mild, less saliva, does not affect eating and speaking; Grade 3: Frequent dry mouth, little and sticky saliva, plenty of water for eating or speaking; Grade 4: Dry mouth causes a burning sensation in the mouth, very little saliva, difficult speech, chewing and swallowing.

(2) Nasal congestion: grade 4, grade 0: none; grade 1: occasional nasal obstruction; grade 2: equivalent, nasal obstruction at or above 2 times a day, affecting sleep; grade 3: extremely persistent nasal obstruction, open mouth for breathing.

(3) Tinnitus: grade 4, grade 0: none; Grade 1: Mild tinnitus, which occurs only in a quiet environment and does not affect sleep; Grade 2: Tinnitus is loud, the general environment can be heard, does not affect the rest; Grade 3: Any environment can be heard, affecting sleep work.

(4) Trismus: grade 5, Grade 0: no change; Grade 1: Limited opening, incisor pitch 2.1–3.0 cm; Grade 2: Difficulty in eating, incisor distance 1.1–2.0 cm; Grade 3: Difficulty in feeding soft food, 0.5–1.0 cm incisor distance; Grade 4: Incisor spacing < 0.5 cm, requiring nasal feeding.

(5) Neck fibrosis: Grade 4, grade 0: no change; grade 1: mild, skin elasticity is poor, pigmentation or decline, and activity is fair; grade 2: moderate, skin inelastic, head rotation < 90 and > 45 ° to the left or right; grade 3: severe, skin plate or/and contracture, head rotation < 45 ° to the left or right.

#### Cancer-related fatigue assessment

It was assessed using the Cancer Fatigue Scale [33]. There were 15 items and 3 dimensions: physical fatigue, emotional fatigue, and cognitive fatigue. All items were scored by Likert’s 5-level scoring method (1 point = none at all, 2 points = very little, 3 points = a little bit, 4 points = quite a lot, 5 points = very much). The total fatigue score range was 0–60 points, 0 points indicated no fatigue state, the higher the score, the more serious the fatigue symptoms, the better the Chinese version of the scale had good credit The Cronbach’s α coefficient of the total scale was 0.88.

#### Quality of life assessment

The QOL of NPC patients was assessed using the EORTC QLQ-H&N35 [3]. The scale includes 7 symptom areas and 35 items in total. Likert 4 rating methods are adopted for each item, and the score ranges from “no” to “persistent existence”. The original score of each field can be obtained by adding the scores of items included in each field and dividing them by the number of items included. Further, the range method is adopted to carry out a linear transformation, and the original score is converted into a standard score of 0 ~ 100 points, to make each field comparative [34]. The higher the score or the more problems in the seven symptom areas, the poorer the overall quality of life. The Cronbach’s coefficient in all fields of the scale is ≥0.70, with good reliability and validity.

### Data collection procedure

The general data, radiation toxicity and side effects, cancer fatigue, and quality of life scores of the patients in both the groups were collected by the members of the continuation nursing team before and after the intervention at 3, 6, and 12 months, respectively.

Telephone follow-ups were utilized for collecting information from the patients in the control group, and platform questionnaires or telephone follow-ups were used for collecting the patients in the intervention group.

### Statistical analysis

The programs Microsoft Excel and IBM statistics SPSS 22.0 were used for data entry as well as statistical description and analysis, respectively. The difference was statistically significant at *P* < 0.05. The data following the normal distribution were expressed as the mean ± standard deviation, and the counting data was expressed by the number of cases (%). The independent-sample t-test and Chi-squared test were performed for single-factor analysis; the repeated measurement analysis of variance was applied to analyze the scores of QOL and cancer-related fatigue, while the nonparametric test was performed for grades of complications of the patients in the two groups. QOL was used as the primary outcome. Cancer-related fatigue and complications were used as the secondary outcomes.

## Result

**Fig. 5:**
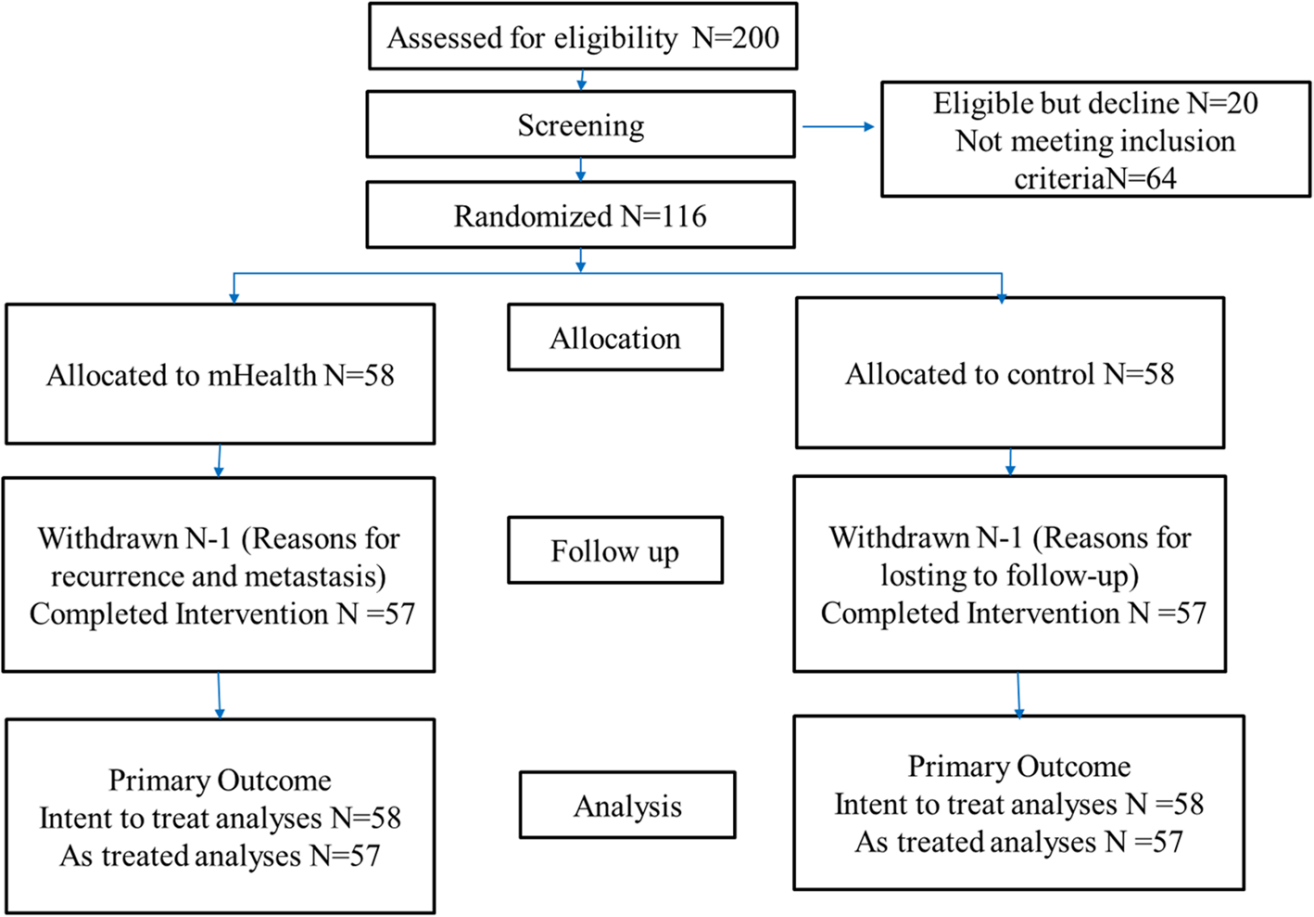
CONSORT

This study involved a total of 116 patients who were randomly allocated, with 58 patients each in the control and intervention groups **(CONSORT** Fig. 5**)**. During the implementation of the project, there was one dropout in the control group due to the loss of follow-up, and one dropout in the intervention group due to distant metastasis. The total dropout rate was 1.72%. Finally, 57 patients in the control and intervention groups completed the trial. This was an intervention study on health education, and it is a non-invasive procedure and non-drug intervention. No serious adverse events related to the mHealth based health education were registered throughout the research process.

### General data of the patients

There was a total of 114 patients comprising 57 in the control group and 57 in the intervention group. The general data characteristics of the two groups were compared using a chi-square test or independent-sample *t-*test compared, and the difference was not statistically significant (*P* > 0.05), which indicated that the two groups were comparable. **(**Table 1**).**

**Table 1:** Baseline characteristics **(***n* ***=*** 114**)**

General situation	Control group	Intervention group	*t*/χ^2^ value	*P****-***value
	*n* ***=*** 57	*n* = 57		
**Age**			1.090	0.580
18-	28 (49.1)	25 (43.9)		
45-	28 (49.1)	30 (52.6)		
60-	1 (1.8)	2 (3.5)		
**Sex**			2.090	0.148
Male	44 (77.2)	37 (64.9)		
Female	13 (22.8)	20 (35.1)		
**Marital status**			0.704	0.402
Unmarried	4 (7.0)	2 (3.5)		
Married	53 (93.0)	55 (96.5)		
Education level			1.880	0.598
Primary school and below	12 (21.1)	17 (29.8)		
Junior high school	28 (49.1)	27 (47.4)		
High school/technical secondary school	7 (12.3)	7 (12.3)		
College or above	10 (17.5)	6 (10.5)		
**Monthly household income**			0.799	0.671
<3000 yuan	37 (64.9)	40 (70.2)		
3000–5000 yuan	16 (28.1)	12 (21.0)		
>5000 yuan	4 (7.0)	5 (8.8)		
**The payment method for medical expenses**			1.614	0.204
New rural cooperative medical system (NCMS)	43 (75.4)	42 (73.7)		
Urban medical insurance (URBMI)	14 (24.6)	15 (26.3)	0.046	0.830
**Family history**				
No	8 (14.0)	6 (10.5)	0.326	0.568
Yes	49 (86.0)	51 (89.5)		
Disease staging			2.527	0.112
Phase II and below	6 (10.5)	6 (10.5)		
Phase III	21 (36.9)	25 (43.9)		
Phase IV	30 (52.6)	26 (45.6)		
Chemotherapy cycle ()	3.58 ± 0.96	3.28 ± 0.86	1.744^1)^	0.084
Treatment plan			0.049	0.862
Induction chemotherapy + concurrent chemoradiotherapy	14 (24.6)	13 (22.8)		
concurrent chemoradiotherapy	43 (75.4)	44 (77.2)		

*Note;*1) as the *t* value.

### Toxicities and side effects of radiotherapy before and after intervention

There was no significant difference between the two groups before the intervention (*P* > 0.05) **(**Table 2**)**. After 3 months of intervention, there was no significant difference in the severity of symptoms like dry mouth, nasal congestion, tinnitus, trismus, and neck fibrosis between the intervention and control groups (*P* > 0.05). After 6 and 12 months of intervention, the severity of the above symptoms like dry mouth, nasal congestion, tinnitus, trismus, and neck fibrosis in the intervention group was lower than those in the control group, and the difference was statistically significant (*P* < 0.05)，**(**Table 3**).**

**Table 2:** Comparison of toxicity and side effects of radiotherapy between the two groups before intervention (*n =* 114)

General situation	Before the intervention		*Z* value	*P-*value
	Control group (*n =* 57)	Intervention group (*n =* 57)		
Nasal congestion			−1.382	0.167
Grade 0	23 (40.4)	19 (33.3)		
Grade 1	18 (31.6)	10 (17.5)		
Grade 2	6 (10.5)	17 (29.8)		
Grade 3	10 (17.5)	11 (19.3)		
Dry mouth			− 0.137	0.891
Grade 0	2 (3.5)	1 (1.8)		
Grade 1	6 (10.5)	4 (7.0)		
Grade 2	18 (31.6)	19 (33.3)		
Grade 3	26 (45.6)	32 (56.1)		
Grade 4	5 (8.8)	1 (1.8)		
Tinnitus			−0.804	0.421
Grade 0	29 (50.9)	23 (40.3)		
Grade 1	7 (12.3)	9 (15.8)		
Grade 2	11 (19.3)	16 (28.1)		
Grade 3	10 (17.5)	9 (15.8)		
Trismus			−1.531	0.126
Grade 0	45 (79.0)	51 (89.4)		
Grade 1	10 (17.5)	5 (8.8)		
Grade 2	2 (3.5)	1 (1.8)		
Neck fibrosis			−0.540	0.590
Grade 0	46 (80.7)	48 (84.2)		
Grade 1	6 (10.5)	6 (10.5)		
Grade 2	5 (8.8)	3 (5.3)

**Table 3:** Comparison of toxicity and side effects of radiotherapy between the two groups after intervention (*n =* 114)

Project	grading	Three months after the intervention		*Z* value	*P*-value	Six months after the intervention		*Z* value	*P*-value	Twelve months after the intervention		*Z* value	*P*-value
		Control group	Intervention group			Control group	Intervention group			Control group	Intervention group		
Nasal congestion	Grade 0	18 (31.6)	20 (35.1)	−0.310	0.757	22 (38.6)	33 (57.9)	−2.510	0.012	24 (42.1)	36 (63.2)	−2.735	0.006
	Grade 1	14 (24.6)	14 (24.6)			16 (28.1)	16 (38.1)			18 (31.6)	17 (29.8)		
	Grade 2	20 (35.0)	18 (31.6)			17 (29.8)	8 (14.0)			13 (22.8)	4 (1.0)		
	Grade 3	5 (8.8)	5 (8.8)			2 (3.5)	0 (0.0)			2 (3.5)	0 (0.0)		
Dry mouth	Grade 0	3 (5.3)	4 (7.0)	−1.322	0.186	5 (8.8)	8 (14.0)	−2.540	0.011	15 (26.3)	20 (35.1)	−2.430	0.015
	Grade 1	9 (15.8)	7 (12.3)			17 (29.8)	24 (42.1)			22 (38.6)	29 (50.9)		
	Grade 2	22 (38.6)	33 (57.9)			21 (36.8)	23 (40.4)			11 (19.3)	6 (10.5)		
	Grade 3	23 (40.3)	13 (22.8)			14 (24.6)	2 (3.5)			9 (15.8)	2 (3.5)		
	Grade 4	0 (0.0)	0 (0.0)			0 (0.0)	0 (0.0)			0 (0.0)	0 (0.0)		
Tinnitus	Grade 0	31 (54.4)	25 (43.8)	−0.493	0.622	29 (50.9)	40 (70.2)	− 2.116	0.034	32 (56.1)	43 (75.4)	−2.080	0.037
	Grade 1	6 (10.5)	13 (22.8)			15 (26.3)	11 (19.3)			17 (29.8)	13 (22.8)		
	Grade 2	15 (26.3)	16 (28.1)			11 (19.3)	3 (5.2)			6 (10.5)	1 (1.8)		
	Grade 3	5 (8.8)	3 (5.3)			2 (2.5)	3 (5.3)			2 (3.5)	0 (0.0)		
Trismus	Grade 0	45 (78.9)	51 (89.4)	−1.505	0.132	42 (73.7)	52 (91.2)	−2.405	0.016	44 (77.2)	53 (93.0)	−2.295	0.022
	Grade 1	11 (19.3)	5 (8.8)			14 (24.5)	4 (7.0)			13 (22.8)	3 (5.2)		
	Grade 2	1 (1.8)	1 (1.8)			1 (1.8)	1 (1.8)			0 (0.0)	1 (1.8)		
Neck fibrosis	Grade 0	39 (68.4)	47 (82.4)	−1.667	0.095	35 (61.4)	47 (82.4)	−2.471	0.013	38 (66.7)	50 (87.7)	−2.638	0.008
	Grade 1	14 (24.6)	7 (12.3)			20 (35.1)	9 (15.8)			17 (29.8)	6 (10.5)		
	Grade 2	4 (7.0)	3 (5.3)			2 (3.5)	1 (1.8)			2 (3.5)	1 (1.8)		

### The cancer-related fatigue score before and after the intervention

There was no significant difference in the CRF score between the two groups before the intervention (*P* > 0.05). After 6 and 12 months of intervention, the scores of total fatigue, physical fatigue, emotional fatigue, and cognitive fatigue in the two groups were lower than those in the control group, with a statistical significance (*P* < 0.05). The results of the repeated measurement ANOVA with two samples showed that, extending the intervention time significantly reduced cancer in the two groups than that in the control group (*P* < 0.05). After 6 and 12 months of intervention, the score of the cancer fatigue in the intervention group was lowered than that in the control group (*P* < 0.05) **(**Table 4**)**.

**Table 4:** Comparison of cancer fatigue between the two groups before intervention (*n =* 114)

Project	Before intervention	Six months after the intervention	Twelve months after the intervention	*F* value	*P*^*2*^ value
The total fatigue					
Control group	26.46 ± 4.21	24.09 ± 3.21	22.61 ± 2.91	*F*_1_ = 39.297	<0.001
Intervention group	26.65 ± 3.51	19.86 ± 3.63	15.75 ± 3.08	*F*_2_ = 18.475	<0.001
*t* value	−0.266	−6.586	−12.247	*F*_3_ = 189.465	<0.001
*P*^*1*^ value	0.791	<0.001	<0.001		
Physical fatigue					
Control group	10.82 ± 3.10	9.91 ± 2.54	9.21 ± 2.45	*F*_1_ = 9.371	<0.001
Intervention group	9.73 ± 3.18	7.63 ± 2.26	6.14 ± 1.90	*F*_2_ = 6.311	0.015
*t* value	−1.848	−5.055	−7.476	*F*_3_ = 55.863	<0.001
*P*^*1*^ value	0.067	<0.001	<0.001		
Emotional fatigue					
Control group	8.94 ± 1.24	8.08 ± 1.53	7.684 ± 1.48	*F*_1_ = 14.537	<0.001
Intervention group	9.28 ± 1.57	7.31 ± 1.66	6.070 ± 1.57	*F*_2_ = 5.650	0.021
*t* value	1.257	−2.575	−5.677	*F*_3_ = 79.988	<0.001
*P*^*1*^ value	0.211	0.011	0.003		
Cognitive fatigue					
Control group	6.87 ± 1.56	5.67 ± 1.73	5.71 ± 1.44	*F*_1_ = 32.947	<0.001
Intervention group	6.94 ± 1.65	4.91 ± 1.47	3.52 ± 1.50	*F*_2_ = 8.819	0.004
*t* value	0.233	−2.506	−7.936	*F*_3_ = 92.185	<0.001
*P*^*1*^ value	0.816	0.014	<0.001		

*Note*: *F1* is the time effect, *F2* is the intergroup effect, *F3* is the interaction effect of time and grouping, *P*^*1*^ represents the result obtained using an independent sample t-test, *P*^*2*^ represents the result obtained by repeated ANOVA

### The quality of life symptom domain scores before and after the intervention

The symptom domains of quality of life between the two groups showed no significant difference in the scores before and after the intervention (*P* > 0.05). After 6 months of intervention, the scores of head and neck pain, swallowing function, sensory problems, and language problems were found to be lowered in the intervention group than those in the control group (*P* < 0.05), while there was no significant difference in the other symptom scores (*P* > 0.05). After 12 months of intervention, the scores of symptom domains were found to be lower in the intervention group than those in the control group (*P* < 0.05); using the two-sample repeated measurement ANOVA, the results showed that except for the language problems, the scores of each symptom domain of quality of life lowered in the intervention group than those in the control group after 6 months and 12 months of intervention, and the scores of symptom domains of the quality of life in the intervention group were found to decrease with the extension of the intervention time, **(**Table 5**)**.

**Table 5:** Comparison of quality of life in various fields between the two groups before intervention (*n =* 114)

Project	Before intervention	Six months after the intervention	Twelve months after the intervention	*F value*	*P*^*2*^ *value*
**Head and neck pain**					
Control group	11.69 ± 5.19	8.91 ± 4.94	8.04 ± 4.86	*F*_1_ = 14.758	<0.001
Intervention group	11.10 ± 6.16	6.72 ± 4.83	6.13 ± 4.86	*F*_2_ = 12.574	0.001
*t* value	−0.548	−2.393	−2.063	*F*_3_ = 13.549	<0.001
*P*^*1*^ value	0.585	0.018	0.041		
**Swallowing function**					
Control group	24.26 ± 10.71	19.90 ± 8.12	18.58 ± 6.67	*F*_1_ = 0.278	0.758
Intervention group *t* value	20.90 ± 7.73	16.23 ± 7.93	14.33 ± 5.39	*F*_2_ = 4.586	0.037
	−1.921	−2.437	−3.745	*F*_3_ = 24.297	<0.001
*P*^*1*^ value	0.057	0.016	<0.001		
**Sensory problems**					
Control group	27.63 ± 15.03	25.15 ± 11.73	14.62 ± 6.91	*F*_1_ = 2.205	0.120
Intervention group *t* value	28.95 ± 15.51	15.93 ± 8.95	10.96 ± 5.02	*F*_2_ = 4.951	0.030
*t* value	0.460	−4.713	−3.230	*F*_3_ = 41.149	<0.001
*P*^*1*^ value	0.647	<0.001	0.002		
**Feeding problems**					
Control group	18.27 ± 8.83	14.47 ± 7.96	15.20 ± 5.48	*F*_1_ = 0.280	0.757
Intervention group*t* value	16.67 ± 10.45	12.71 ± 6.12	10.09 ± 3.77	*F*_2_ = 5.691	0.020
*t* value	−0.888	−1.320	−5.806	*F*_3_ = 22.426	<0.001
*P*^*1*^ value	0.376	0.190	<0.001		
**Language problem**					
Control group	14.03 ± 8.54	12.86 ± 5.86	11.89 ± 4.63	*F*_1_ = 0.926	0.402
Intervention group *t* value	13.45 ± 7.78	9.74 ± 4.24	8.17 ± 4.94	*F*_2_ = 0.363	0.549
*t* value	−0.382	−3.257	−4.132	*F*_3_ = 16.150	<0.001
*P*^*1*^ value	0.703	0.001	<0.001		
**Social problem**					
Control group	11.57 ± 3.89	7.84 ± 3.12	11.11 ± 3.64	*F*_1_ = 3.072	0.054
Intervention group*t* value	11.11 ± 3.63	7.49 ± 3.58	7.48 ± 3.58	*F*_2_ = 8.508	0.005
*t* value	−0.664	− 0.557	−4.184	*F*_3_ = 78.730	<0.001
*P*^*1*^ value	0.508	0.579	<0.001		
**Sexual function**					
Control group	35.67 ± 13.88	33.33 ± 15.43	31.58 ± 15.32	*F*_1_ = 5.344	0.008
Intervention group*t* value	36.25 ± 13.03	29.82 ± 13.26	21.05 ± 13.18	*F*_2_ = 4.505	0.038
*t* value	0.232	−1.302	−3.930	*F*_3_ = 20.030	<0.001
*P*^*1*^ value	0.817	0.196	<0.001		

*Note*: *F1* is the time effect; *F2* is the intergroup effect; *F3* is the interaction effect of time and grouping; *P*^*1*^ represents the result obtained using an independent sample *t*-test； *P*^*2*^ represents the result obtained by repeated ANOVA

## Discussion

### The construction of the mHealth platform is of great significance for the home-based management of NPC patients

NPC as a chronic disease is highly prevalent in the old, rural, border, and poor areas of Guangxi [35]. Due to the long-distance or economic conditions, the patients, especially those in the rural areas, fail to achieve timely professional help and guidance from the medical personnel after discharge. Dai has also proved that discharged patients are more willing to receive professional guidance or one-to-one nursing service without leaving their homes [36]. Therefore, it is imperative to innovate the form of continuing nursing and develop diversified service modes. The mobile internet technology has introduced a brand-new improvised mode for stay-at-home and instant medical care, promoting medical knowledge sharing and doctor-patient or nurse-patient interaction, improving the level of hospital clinical nursing information construction at a deeper level, and diversifying the work of nurses [37]. Chinese scholars have developed the mHealth nursing platform and applied it to manage the health of diabetes, bone arthroplasty, and enterostomy patients [38]. However, the application and research of mHealth-based continuing care in patients with NPC are rare and insufficient in China. Therefore, this study conforms to the development needs of the times for building a continuous care management platform for the NPC patients with functions such as questioning, video playback, graphic editing, knowledge push, questionnaire statistics, and message reminders, which would provide the NPC patients with “immediate care without leaving home” services and overcome the limitations of current community conditions and the shortcomings of the hospital services. Up to now, there are 7 registered communities of the NPC continuous care platform with 19 medical staff, where the utilization rate has reached 98%. The doctor-patient or nurse-patient interactions summed up to be more than 300 times, and the cumulative reading volume was more than 2280 times, indicating the establishment of the internet platform for NPC. It not only innovates the continuous nursing method, by building an interactive platform between the medical staff and patients after the hospital, but more importantly accelerates the dissemination of health information, improving the level of information-based nursing services, as well as patient satisfaction, and benefits the majority of patients. It also indirectly illustrated the desire of the patients subjected to home rehabilitation for gathering knowledge about disease and health and the lack of access to the previous channels. Therefore, future studies should reinforce the combination of information technology and medical care, for continuously standardizing and innovating the form of continuous management.

### The application of the mHealth management can reduce the incidence of toxic and side effects

The toxic and side effects of radiotherapy were found to be alleviated by effective nursing. This study showed that the application of mHealth to carry out continuous nursing services can reduce the incidence of symptoms like nasal congestion, dry mouth, tinnitus, neck fibrosis, and difficulty in the opening mouth (*P* < 0.05). Related studies have also confirmed that mobile health applications might improve aspects of symptom control in patients with cancer [6, 39]. Thus, the mHealth platform might be applicable for enlightening the patients with advanced knowledge of nursing, through dynamic video, and personalized counseling, that would effectively cater to the needs of the patients in getting the nursing service and urge the patients to take effective nursing measures according to the inquiry function to improve their compliance and self-care skills, and finally reducing the symptoms like dry mouth, tinnitus, nasal congestion, and neck in the mouth. The degree of the side effects such as fibrosis and difficulty in opening the mouth were also taken into consideration.

### The mHealth management can reduce the degree of cancer-related fatigue

Studies have shown gender, chemoradiotherapy, depression, pain, and sleep disorders to be closely related to CRF in NPC patients. Appropriate exercises can effectively improve the degree of CRF, and high compliance aerobic exercises have been found to show better efficacy than resistance exercises [40]. Other studies have demonstrated that therapies assisting the patients in recognizing and correcting the erroneous ideas and behaviors, starting with the triggers of negative symptoms, have a significant clinical effect in reducing the CRF and functionally impairing the cancer survivors [41, 42]. This study showed that mHealth can be implemented for improving the CRF of patients at home by extending the care services in a highly significant manner. This might be because the service is based on the Internet platform providing a convenient condition for professional consultation and a good environment for emotional communication. It promotes the establishment of a virtuous circle for mutually improving the somatic symptoms and negative emotions. During the consultation, the medical staff solved the patients’ doubts, providing methods to improve the symptoms and change their cognition. Through communication, the medical staff could understand the patients’ false cognition, carry out the targeted intervention, reconstruct the patients’ cognitive concepts, and improve the patients’ sense of fatigue. In addition, the head and neck exercise belonging to the aerobic exercises affects by reducing the patients’ sense of CRF. Online communication with the patients using the internet platform not only provides nursing methods and supervision to the patients but also provides emotional and information support, for changing the patients’ cognition and reducing fatigue.

### The mHealth application can improve the QOL of patients

QOL is an important component for evaluating the health care system, and the primary goal of implementing continuous nursing services is to improve the quality of life of the tumor patients at home. The 5-year survival rates of NPC patients have reached more than 80% [43]. However, the chronic specific symptoms, such as head and neck pain, swallowing function, sensory problems, language problems, difficulty in eating, dry mouth, viscous saliva, and a variety of derived negative emotions, such as depression and anxiety, make the survivors of NPC to further suffer from physical and mental suffering, hampering the QOL [44]. This research is based on the mHealth to carry out continual nursing for NPC. The results showed that the scores of the 7 symptom areas of pain, swallowing, sensation, eating, language, social contact, and sexual function in the observation group were all lower than those in the control group after intervention (*P* < 0.05). In other words, the QOL of patients based on the extended care of the network platform is better than the traditional follow-up management. This is similar to the research on applying “Internet +” in the chronic disease management model for type 2 diabetes by Yun [45], and the results of Dai in designing and applying the networked management system for stroke patients [46]. The reasons might be that: (1), Combining the continuity of care with the “Internet +” to carry out the network management service would overcome the space and time constraints, and achieve seamless guidance and supervision, conducive to improving the health outcomes of the patients and improving the QOL of the patients. (2) The information management and emotional support can assist the patients in performing rehabilitation nursing and functional exercise, and that would reduce the degree of various specific symptoms in the patients. The internet platform is always the carrier, and the most important factor for promoting the rehabilitation of the patients is the pertinence, rationality, and standardization of the content. (3) The “Internet +” continuity of care can effectively prevent the patients from visiting the hospitals frequently, reducing the cost of medical services as well as the burden. (4) The continuity of care based on the “Internet +” is extended to the home, achieving the continuity of information, management, and relationship.

### Limitations

This study was a single-center study, and the number of included cases was relatively small. One of the inclusion criteria of the study subjects was the ability to use smartphones, and the platform was developed in Chinese. These limitations might affect the study results and generalized applications. In the future, the application of the platform can be further promoted and multi-center, large-sample application research should be performed for improving the credibility of the research. Moreover, considering this intervention for all stages of the disease it can be recommended for developing and supporting different languages, such as English, for future studies.

## Conclusion

This study provided professional, continuous, and overall extended care for the discharged NPC patients by constructing the mHealth platform and implementing a continuous nursing scheme based on the patient’s needs. The mHealth-based nursing model can reduce the toxic and side effects of radiotherapy and cancer fatigue in patients with nasopharyngeal cancer, and improve the quality of life, proving to be a more suitable method for long-term chronic management. This study can provide a reference for follow-up management of all the patients with chronic diseases.

## Data Availability

The data that support the findings of this study are not openly available due to human data and are available from the corresponding author upon reasonable request.

## Abbreviations

mHealth: Mobile Health
NPC: Nasopharyngeal carcinoma
QOL: The quality of life
CRF: Cancer-related fatigue
